# Comparison of SYNTAX and Gensini Scores in the Decision of Surgery or Percutaneous Revascularization in Patients With Multivessel Coronary Artery Disease

**DOI:** 10.7759/cureus.22482

**Published:** 2022-02-22

**Authors:** Bedrettin Boyraz, Tezcan Peker

**Affiliations:** 1 Cardiology, Private Doruk Yildirim Hospital, Bursa, TUR

**Keywords:** coronary artery by-pass grafting, percutaneous coronary intervention, revascularization decision, gensini score, syntax score, multivessel coronary artery disease (mvcad)

## Abstract

Introduction: Many scoring systems have been developed to determine the extent of coronary artery disease (CAD) in patients with multi-vessel disease. The most widely used of these are Synergy between percutaneous coronary intervention with Taxus and cardiac surgery (SYNTAX) and Gensini scoring. Gensini scoring system can successfully show coronary plaque burden. In our study, we aimed to test the predictive power of SYNTAX and Gensini scores for surgical or percutaneous intervention decisions made by the cardiac team in patients with three-vessel disease.

Methods: A total of 476 patients without ST-elevation myocardial infarction with the three-vessel disease were included in the study. SYNTAX and Gensini scores of the patients were calculated from their coronary angiographies. Receiver operating characteristic curve (ROC-curve) analysis was performed using both scores.

Results: Both the SYNTAX score and Gensini score were able to predict heart team decisions (AUC: 0.759, 0.680; p<0.001). Diabetes and smoking were more common in patients who were decided to have surgery (p<0.001).

Conclusion: In the light of our study results, the decisions to be made with the SYNTAX score will be closer to the decisions of the heart team than the Gensini score.

## Introduction

Although it is an invasive method in the diagnosis of coronary artery disease (CAD), coronary angiography (CAG) is the gold standard method [1]. Many scoring systems have been developed that allow the extent of plaques and stenoses observed as a result of CAG and the severity of CAD to be expressed numerically. The most widely used of these is the Synergy between percutaneous coronary intervention with Taxus and cardiac surgery (SYNTAX) score. The SYNTAX score is a scoring system that is successful in showing the extent and severity of the CAD, as well as providing valuable information in terms of prognosis and the choice of revascularization method [2]. Current guidelines recommend the use of the SYNTAX score for the choice of surgical revascularization (CABG) or percutaneous revascularization (PCI) treatment [3]. Another widely used scoring system is the Gensini scoring system. Many studies are showing that the Gensini score system provides valuable information in terms of prognosis and predicts the prevalence of coronary plaque [4]. One of the biggest differences between the two scoring systems is that coronary plaques with less than 50% narrowing are not evaluated in the SYNTAX scoring system, but these lesions are included in the Gensini scoring system [5,6]. This situation is also important in the selection of revascularization treatment because, in the case of revascularization, procedures are performed for lesions that cause stenosis of 70% or more. In addition to all these conditions, the degree and score of the stenosis alone do not determine the choice of the revascularization method. The extent of the lesion or lesions and the suitability of the lesions for PCI or CABG also play a decisive role. Although these scoring systems are guiding in the choice of treatment, guidelines recommend that the heart team decide [7]. Based on all these data, we planned our study to determine which system can better predict treatment choices and which system is more useful in making decisions on this issue in patients whose three-vessel disease in CAG was determined by the cardiac team, and for whom CABG or PCI was decided.

## Materials and methods

All study procedures involving human participants were in accordance with the ethical standards of the institutional and national research committee and with the 1975 Helsinki declaration and its later amendments or comparable ethical standards. Ethics committee approval was obtained from the scientific research ethics committee of the University of Health Sciences, Diyarbakir Gazi Yaşargil Education and Research Hospital, dated October 8, 2021; Approval number: 912.

Study design

The study is a retrospective, observational, cross-sectional study. Our hospital is the only heart center in its region. PCI and CABG decisions are made according to the joint decision of the heart team. Parameters such as the existing comorbidities of the patients, the burden of CAD, and their suitability for revascularization methods are important in this decision. A specific scoring system is not adhered to. The files of the patients who underwent CAG between 2017-2019 in our hospital were examined. Among the patients with three-vessel CAD, those who presented to our hospital with ST-elevation myocardial infarction or had a previous CABG history were excluded. The patients were divided into two groups: stable angina pectoris (SAP) and acute coronary syndrome (ACS) patients. The ACS group consisted of non-ST elevation myocardial infarction and unstable angina pectoris patients. Demographic data such as CAD history, hypertension, diabetes, hyperlipidemia, age, gender, and smoking were obtained from the patients' files and recorded. Previously performed CAGs of the patients were analyzed and SYNTAX and Gensini scores were calculated [3,4].

Statistical analysis

In statistical analysis, we performed analysis using chi-square and Fisher’s exact test for categorical variables and Student’s t-test for continuous variables. Categorical variables were expressed in numbers and percentages, continuous variables are expressed as mean± SD. Receiver operating characteristic curve (ROC-curve) analysis of SYNTAX and Gensini scores were performed in terms of revascularization methods. Data are summarized with area under the curve (AUC) values, 95% CI, and p-value. IBM SPSS Statistics for Windows, Version 22.0 (Released 2013. IBM Corp., Armonk, New York, United States) and Stata Statistical Software: Release 17 (2021, StataCorp LLC, College Station, Texas, United States) were used in statistical analysis.

## Results

A total of 476 patients who underwent CAG due to non-ST segment elevation myocardial infarction (non-STEMI) ACS and SAP and who were found to have the three-vessel disease were evaluated. The demographic data of the patients, SYNTAX scores, and Gensini scores are summarized in Table *1* 

**Table 1:** Demographic and score parameters Categorical parameters are expressed as numbers (percentages), and continuous variables are expressed as mean (SD) CABG: coronary artery bypass grafting; PCI: percutaneous coronary intervention; SYNTAX: synergy between percutaneous coronary intervention with taxus and cardiac surgery

	Total	PCI	CABG	p-value
Patients	476	294 (61.8%)	182 (38.2%)	< 0.001
Age	64.29 (11.36)	64.87 (11.78)	63.35 (10.61)	0.15
Gender (Male)	330 (69.3%)	206 (70.1%)	124 (68%)	0.68
Coronary artery disease	98 (20.6%)	71 (24.1%)	27 (14.8%)	0.015
Hypertension	223 (46.8%)	145 (49.3%)	78 (42.9)	0.18
Diabetes mellitus	222 (46.6%)	96 (32.7%)	126 (69%)	< 0.001
Hyperl ipidemia	226 (47.5%)	141 (48%)	85 (46.7%)	0.85
Smoke	180 (37.8%)	92 (31.3%)	88 (48%)	< 0.001
SYNTAX score	24.73 (7.30)	22.30 (5.78)	28.66 (7.79)	< 0.001
Gensini score	71.74 (28.11)	64.86 (25.03)	82.86 (29.29)	< 0.001

**Figure 1:**
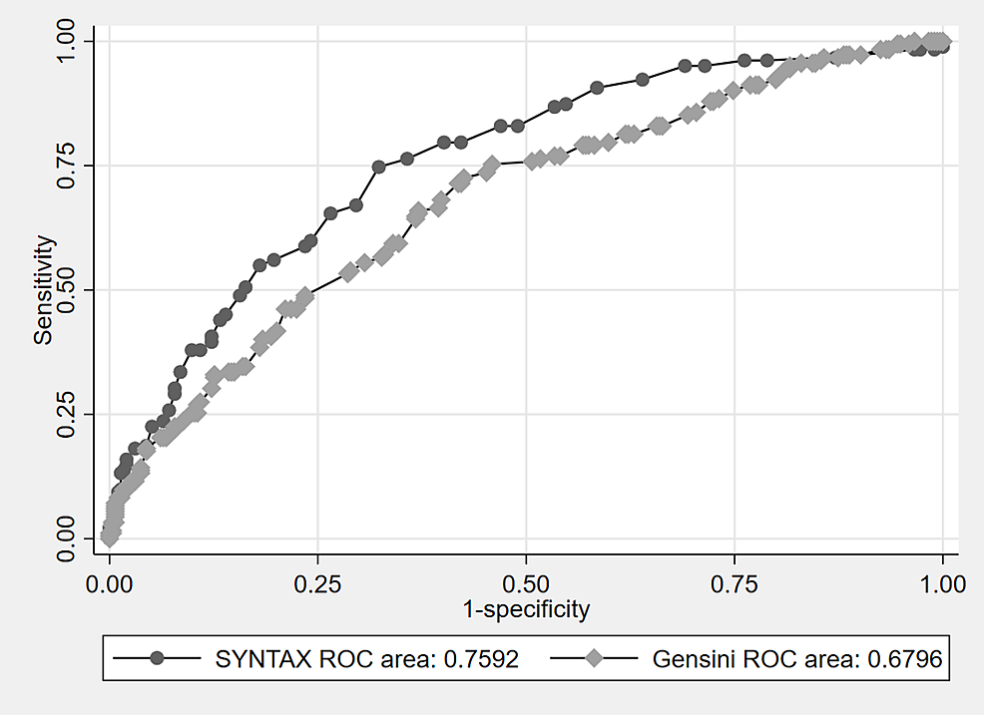
ROC-curve analysis SYNTAX 95% confidence interval: 0.715-0.803; Gensini 95% confidence interval: 0.630-0.729 SYNTAX: synergy between percutaneous coronary intervention with taxus and cardiac surgery; ROC: receiver operating characteristic

The rate of patients who underwent CABG after CAG for SAP was found to be significantly higher than the patients admitted for ACS (71 (60%), 111 (31%), respectively; p<0.001). There was no significant difference between the patients in terms of SYNTAX and Gensini scores according to the admission clinics (p: 0.57, 0.69, respectively). In the analysis made according to gender, the average age of female patients was found to be higher than men (68.15±10.45, 62.58±11.34, respectively; p<0.001). ROC-curve analysis was performed between both systems according to the revascularization decision. The SYNTAX score was found to be significantly superior to the Gensini score (AUC: 0.759, 0.680; p: <0.001, respectively) (Figure 1).

## Discussion

According to our study results, both scoring systems can successfully predict the heart team's choice of revascularization method, and the SYNTAX score can predict the decision better than the Gensini score. Although both systems can predict angiographically and anatomically coronary plaque burden and the extent of occlusive CAD, the difference was thought to be the main reason because the coronary revascularization procedure was aimed at occlusive CAD with a rate of 70% or more. Although small studies are showing that the Gensini score system can predict coronary plaque burden better than the SYNTAX score, it was found to be more unsuccessful in the selection of coronary revascularization compared to the SYNTAX score in our study. In studies evaluating coronary plaque burden, short and long-term prognoses were the main point rather than the treatment needs of the patients [4]. Charach et al. conducted a study in which they concluded that the Gensini score could be a useful scoring system in angiographically detecting the severity of coronary artery disease, showing prognosis, and predicting possible benefits from treatments [8]. Wang et al. indicated in their study that the Gensini score was an independent predictor of long-term adverse outcomes in patients with CAD who underwent PCI, and it had more predictive value in the population with diabetes [9]. The lack of follow-up data in our study is the main limitation of our study. However, our study design and the fact that our study aims to measure the ability of scoring systems to predict treatment selection rather than predicting prognosis distracts us from the possibility that this will adversely affect our study results. If we look at its use in basic current practice, determining the prevalence of coronary plaque burden will not cause any change in both medical and interventional treatment. In patients with obstructive CAD, drug treatments with indications are recommended according to the presence or absence of the disease rather than the extent of the disease, while interventional treatments are given according to these scoring systems, the patient's clinic, and the decisions of the heart team.

These scoring systems and angiographically-detected lesion prevalence are not the only determinants in the choice of revascularization method. The existing comorbid conditions of the patients are effective in the selection of the revascularization method. Diabetes is a well-known risk factor for CAD. Both the prevalence and frequency of CAD increase in patients with diabetes compared to patients without diabetes. In many studies, it has been shown that CABG treatment in patients with DM and three-vessel disease gives better results in terms of prognosis of patients compared to PCI [10,11]. In our study, in support of these findings, DM was significantly higher in patients who were given a CABG decision than the group in which PCI was decided. Smoking was found to be significantly higher in patients who were given CABG decisions. Smoking is one of the known correctable CAD risk factors. This was thought to be because smoking increases both the prevalence of CAD and coronary artery lesions that need revascularization [12]. There was no difference between the two groups in terms of gender, but the number of men with the three-vessel disease was significantly higher than the number of women, this is because male gender is a known risk factor for CAD [13]. There was no significant difference in age between the two groups, but the age of female patients was found to be significantly higher than male patients. This is in agreement with the known literature [14]. When the choice of revascularization decision is evaluated according to the clinic of admission, it is seen that the CABG decision is made significantly more frequently in SAP patients than in ACS patients. It was thought that PCI treatment should be preferred more in order not to delay the revascularization treatment in cases such as the arrhythmic or hemodynamic condition is unstable. This situation is also striking in the multi-registry study of Freitas et al. In the study, the median time from hospital admission to CABG treatment was found to be nine days. Based on this, they indicate that the patients in the CABG group were lower-risk patients [15].

## Conclusions

The SYNTAX score was found to be significantly superior to the Gensini score in predicting the revascularization decision made by the heart team in the patients with non-STEMI ACS and SAP who were found to have the three-vessel disease as a result of CAG. In addition to these scoring systems, diabetes and smoking are among the factors that affect the decision of CABG. In the light of our study results, the decisions to be made with the SYNTAX score will be closer to the decisions of the heart team than the Gensini score. Therefore, it seems reasonable to use the SYNTAX score in the selection of revascularization.
